# Supplementary material for: Identification of photoperiod-induced specific miRNAs in the adrenal glands of Sunite sheep (Ovis aries)
Source: Front Vet Sci. 2022 Jul 22;9:888207. doi: 10.3389/fvets.2022.888207 (PMC9354845; doi:10.3389/fvets.2022.888207)
Supplement: Supplementary file 1 [file Data_Sheet_1.ZIP › Supplementary file/miRDeep2 file.zip!/miRDeep2 file/Know_Structure/LP42A1/oar-let-7d.pdf]

miRBase precursor : oar-let-7d
Total read count : 26177
oar-let-7d read count : 23501
remaining reads : 2676

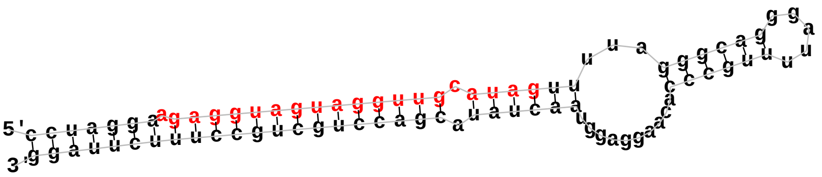

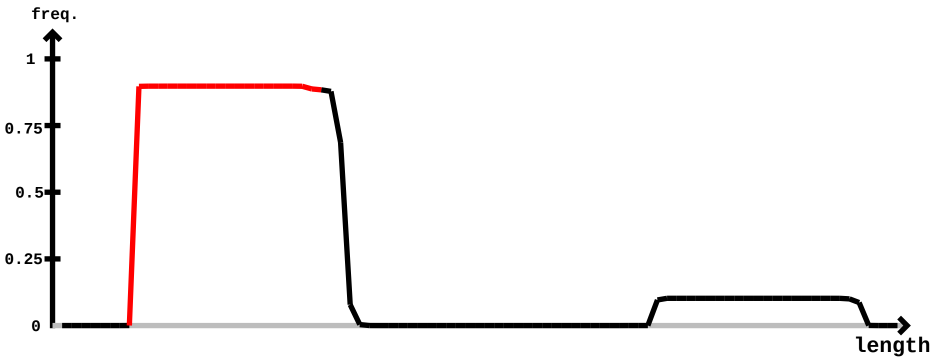


```
          oar-let-7d
5'- ccuaggaagagguaguagguugcauaguuuuagggcagggauuuugcccacaaggagguaacuauacgaccugcugccuuucuuagg  -3'    exp
    ((((((.((((((((((((((.((((((...((((((.....)))))).........)))))).))))))))))))))))))))     reads      mm        sample
    ......aagagguaguagguugcauagu...........................................................  1          0         seq
    ......aagagguaguagguugcauaguu..........................................................  4          0         seq
    .......agagguaguagguugca...............................................................  12         0         seq
    .......agagguaguagguugcau..............................................................  261        0         seq
    .......agagguaguagguuAcaua.............................................................  1          1         seq
    .......agagguaguagguugcaua.............................................................  88         0         seq
    .......agagguaguagguugcauag............................................................  151        0         seq
    .......agagguaguagguugcauagu...........................................................  5004       0         seq
    .......agagguaguagguugcauaguu..........................................................  15902      0         seq
    .......agagguaguagguugcauaguuu.........................................................  1954       0         seq
    .......agagguaguagguugcauaguuuu........................................................  97         0         seq
    .......agagguaguagguugcauaguuuua.......................................................  2          0         seq
    ........gagguaguagguugcau..............................................................  1          0         seq
    ........gagguaguagguugcauagu...........................................................  3          0         seq
    ........gagguaguagguugcauaguu..........................................................  18         0         seq
    .........agguaguagguugcauaguu..........................................................  1          0         seq
    ..........gguaguagguugcauaguu..........................................................  1          0         seq
    ..........................................................uaacuauacgaccugcugccuu.......  1          0         seq
    ..........................................................uaacuauacgaccugcugccuuuc.....  2          0         seq
    .............................................................cuauacgaccugcugcc.........  1          0         seq
    .............................................................cuauacgaccugcugccuu.......  2          0         seq
    .............................................................cuauacgaccugcugccuuu......  51         0         seq
    .............................................................cuauacgaccugcugccuuuc.....  328        0         seq
    .............................................................cuauacgaccugcugccuuucu....  2117       0         seq
    .............................................................cuauacgaccugcugccuuucuu...  12         0         seq
    ..............................................................uauacgaccugcugccuu.......  3          0         seq
    ..............................................................uauacgaccugcugccuuuc.....  30         0         seq
    ..............................................................uauacgaccugcugccuuucu....  128        0         seq
    ..............................................................uauacgaccugcugccuuucuu...  1          0         seq
```
